# Ethics education to support ethical competence learning in healthcare: an integrative systematic review

**DOI:** 10.1186/s12910-022-00766-z

**Published:** 2022-03-19

**Authors:** Henrik Andersson, Anders Svensson, Catharina Frank, Andreas Rantala, Mats Holmberg, Anders Bremer

**Affiliations:** 1 Faculty of Health and Life Sciences, Linnaeus University, Växjö, Sweden; 2 Centre of Interprofessional Collaboration within Emergency Care (CICE), Linnaeus University, Växjö, Sweden; 3 Faculty of Caring Science, Work Life, and Social Welfare, University of Borås, 50190 Borås, Sweden; 4 Department of Ambulance Service, Region Kronoberg, Växjö, Sweden; 5 Department of Health Sciences, Lund University, Lund, Sweden; 6 Emergency Department, Helsingborg General Hospital, Helsingborg, Sweden; 7 Centre for Clinical Research Sörmland, Uppsala University, Eskilstuna, Sweden; 8 Department of Ambulance Service, Region Sörmland, Katrineholm, Sweden; 9 Department of Ambulance Service, Region Kalmar County, Kalmar, Sweden

**Keywords:** Ethical competencies, Ethical problems, Ethics education, Healthcare professionals, Integrative systematic review, Students

## Abstract

**Background:**

Ethical problems in everyday healthcare work emerge for many reasons and constitute threats to ethical values. If these threats are not managed appropriately, there is a risk that the patient may be inflicted with moral harm or injury, while healthcare professionals are at risk of feeling moral distress. Therefore, it is essential to support the learning and development of ethical competencies among healthcare professionals and students. The aim of this study was to explore the available literature regarding ethics education that promotes ethical competence learning for healthcare professionals and students undergoing training in healthcare professions.

**Methods:**

In this integrative systematic review, literature was searched within the PubMed, CINAHL, and PsycInfo databases using the search terms ‘health personnel’, ‘students’, ‘ethics’, ‘moral’, ‘simulation’, and ‘teaching’. In total, 40 articles were selected for review. These articles included professionals from various healthcare professions and students who trained in these professions as subjects. The articles described participation in various forms of ethics education. Data were extracted and synthesised using thematic analysis.

**Results:**

The review identified the need for support to make ethical competence learning possible, which in the long run was considered to promote the ability to manage ethical problems. Ethical competence learning was found to be helpful to healthcare professionals and students in drawing attention to ethical problems that they were not previously aware of. Dealing with ethical problems is primarily about reasoning about what is right and in the patient’s best interests, along with making decisions about what needs to be done in a specific situation.

**Conclusions:**

The review identified different designs and course content for ethics education to support ethical competence learning. The findings could be used to develop healthcare professionals’ and students’ readiness and capabilities to recognise as well as to respond appropriately to ethically problematic work situations.

## Introduction

Healthcare professionals and students undergoing training in healthcare professions are confronted with a variety of ethical problems in their clinical practice. These ethical problems appear as ethical challenges, conflicts, or dilemmas that influence the daily provision of care and treatment for patients [1, 2]. Addressing these problems requires ethical competencies that involve the ethical dimensions of sensitivity, knowledge, reflection, decision making, action, and behaviour [3]. As the future workforce, students need training to effectively deal with ethically problematic situations [4], and experienced professionals need to develop ways to manage ethical problems [5]. Therefore, it is essential for ethics education to support the learning and development of ethical competencies among healthcare professionals and students undergoing training to work in healthcare. In this study, ethics education is referred to educational components with a content of support and learning activities that promote understanding and management of ethical problems. The focus is on ethics education that is carried out at universities and in clinical practice. In conclusion, it would be valuable to first compile the existing knowledge about designs and course content that support ethical competence learning.

## Background

The provision of care is based on patients’ care needs and the complexity of their health conditions; this process is further complicated by the nature of the care environment, which is frequently chaotic and/or unpredictable, with care often being provided under stressful working conditions [6–10]. Healthcare professionals and students in clinical practice are confronted daily with difficult choices and must cope with questions of ‘rightness’ or ‘wrongness’ that influence their decision-making and the quality of the care provided [11, 12]. The underlying reasons for the emergence of ethical problems in everyday healthcare work are multifaceted, unfold over time, and are caused by factors such as a lack of resources, insufficient leadership, hierarchical organisational structures, chaotic work environments, or a lack of competencies [13]. Ethical problems and value conflicts are inherent in clinical practice and do not necessarily mean that healthcare professionals or students have done anything inappropriate or that structures are inadequate. Whatever the cause, ethical problems can lead to conflicts between principles, values, and ways of acting [14]. This, in turn, might lead to compromised moral integrity and generate moral distress [11, 15, 16], as these reactions result from acting or not acting on the basis of one's own sense of right and wrong [17]. At worst, moral distress can lead to moral injury, which occurs as a result of witnessing human suffering or failing to prevent outcomes that transgress deeply held beliefs [18]. Therefore, healthcare professionals and students in clinical practice need to develop their ethical competencies to be prepared for their responsibility and commitment to caring for patients.

The concept of competence is multifaceted and include many things. In this study, competence is viewed as entailing knowledge, skills, and attitudes that are essential when healthcare professionals and students are carrying out their work in clinical practice [19]. Ethical competence contain components such as the capability to identify ethical problems, knowledge about the ethical and moral aspects of care, reflection on one’s own knowledge and actions, and the ability to make wise choices and carefully manage ethically challenging work situations [3]. Ethical competence is essential for the ability to respect the patient’s rights and the quality of care [20, 21]. This means that ethical competence includes not only knowledge of the ethical and moral aspects of care, but it also includes moral aspects of thinking and decision-making. Furthermore, ethical competence is important since it may prevent or reduce moral distress [22].

Healthcare professionals and students in clinical practice need a solid foundation that supports when they are confronted with ethically problematic situations. Care and treatment depend not only on knowledge and skills or acting according to guidelines; they also depend on personal values, beliefs, and ethical orientations [23]. There are various strategies to support and develop the capability to identify and solve ethical problems. [24]. Ethics education is one such way to develop ethical competencies [20]. Simultaneously, ethics education raises questions about the content and teaching methods relevant for clinical practice [25]. While theoretical education via small-group discussions, lectures, and seminars in which ethical principles are applied is quite common [26], an alternative educational method is simulation-based learning [27]. However, there is no evidence to support the determination of the most effective strategy to promote the application of ethics in care. There are also challenges to teaching and assessing ethics education. For example, ethics education does not always occur contextually or in a realistic situation, and theoretical knowledge of ethics does not necessarily lead to improved ethical practice [28]. Teaching ethical principles and maintaining codes of ethics without contextualising them risks forcing healthcare professionals and students in clinical practice to adapt to ethical practice without questioning their own beliefs. Thus, ethical competence risks being hampered by limited reflection and moral reasoning about the situation as a whole [29].

In summary, ethical problems in everyday healthcare work arise for many reasons, and sometimes themselves constitute threats to ethical values. Hence, healthcare professionals and students in clinical practice require readiness and the capability to recognise and respond appropriately to ethically problematic work situations. Therefore, the aim of this integrative review was to explore the available literature on ethics education that promotes ethical competency learning for healthcare professionals and students undergoing training in healthcare professions.

## Methods

### Design

This integrative review followed the method described by Whittemore and Knafl and was used to summarise and synthesise the current state of research on a particular area of interest [30], which in this study was the area of ethics education in healthcare.

### Procedures

According to Whittemore and Knafl [30], the review process is composed of the following stages, which were applied in this study:

#### Stage 1: problem identification

Two questions were addressed in this review to explore the available literature regarding ethics education: (1) How can ethics education support the understanding and management of ethical problems in clinical practice? (2) What kind of design and course content can support ethical competence learning?

#### Stage 2: literature search

**Fig. 1:**
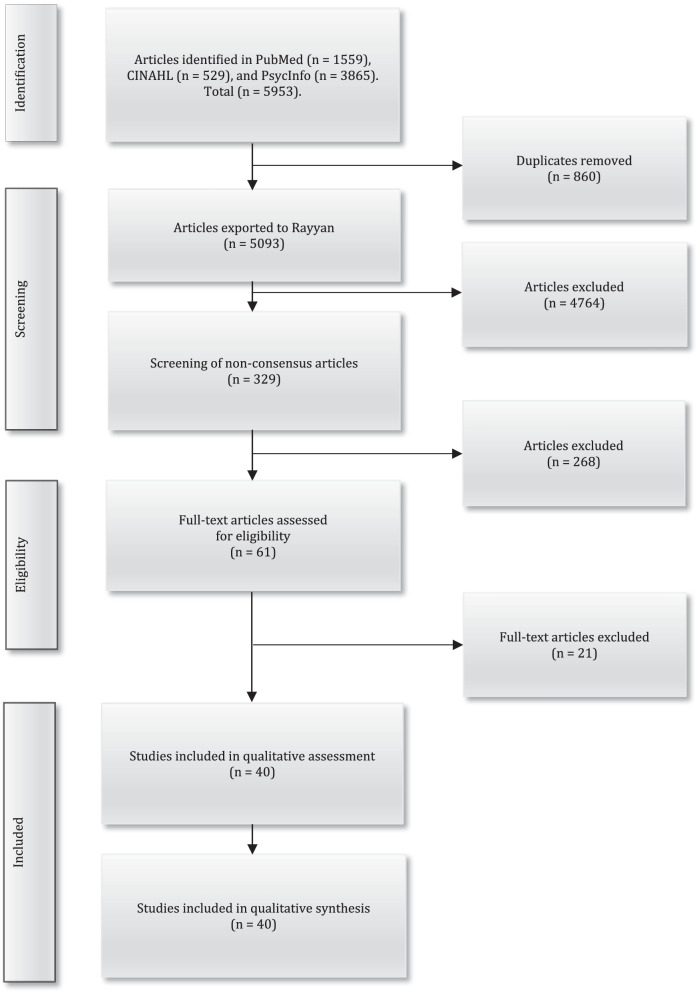
Flow diagram of the data selection and quality assessment process based on the PRISMA statement

Prior to the literature search, a study protocol was submitted to the PROSPERO database with the ID number CRD42019123055. In collaboration with three experienced information specialists at a university library, guidance and support were provided in the creation of a search strategy. A systematic and comprehensive data search was conducted using the standards of the PRISMA guidelines [31]. To enhance the breadth and depth of the database searches, the main search strategy was based on three themes; study population, exposure/intervention and outcomes. The following search and/or Medical Subject Heading’s (MeSH) terms were used: ‘health personnel’, ‘students’, ‘ethics’, ‘moral’, ‘simulation’ and ‘teaching’. The search strategy was different between the databases as the construction of search and MeSH terms differs between the selected databases, see Table 1. The main search was carried out between 22 and 23 June 2020 in three scientific publication databases and indexing services: PubMed, CINAHL, and PsycINFO. A supplementary search was carried out 10 January 2022.The searches was limited to (a) articles in English, (b) peer-reviewed articles, (c) theoretical articles as well as qualitative and quantitative empirical research articles, and d) articles published in the last 12 years (January 1, 2010–December 31, 2021). Articles were included if published after 2010, and they (a) described the design and content of ethics education for healthcare professionals or students in, or preparing for, clinical practice, and/or (b) described ethics education supportive of understanding and/or managing ethical problems in clinical practice. Articles were excluded if they focused on research ethics, ethical problems in a military context and ethical consultation with the primary and main goal of supporting ethical decision-making for an individual patient and the healthcare team. In the literature search, the search for “grey literature” such as dissertations, conference papers, reports, etc. was excluded since this was too resource and time consuming. The article searches resulted in 5953 articles, including 1559 in PubMed, 529 in CINAHL, and 3865 in PsycINFO. For a detailed description of the search results, see Fig. 1. After the search process was completed, all the articles were uploaded onto Endnote X9 (Clarivate Analytics, Philadelphia, PA), and duplicates were then excluded (n = 860). A total of 5093 articles were then imported into the Rayyan QCRI, a web-based sorting tool for systematic literature reviews [32].

**Table 1:** Description of the search strategy with three themes and the search results

Themes	Database, date of search, and search terms	Number of articles Main search	Number of articles Supplementary search
	*PubMed* Main search 2020-06-22 Supplementary search 2022-01-10		
# 1	Health personnel OR Students, health occupations	566,566	29,523
# 2	Ethics OR Ethics professional OR Morals OR Moral	169,269	23,028
# 3	Patient simulation OR Teaching OR Teaching methods OR Teaching methods, clinical OR Learning methods OR Education OR Learning	1,434,299	222,356
# 4	# 1 AND # 2 AND # 3	561	998
Search limiters	Main search: English, abstract, free full text, full text, time limit 2010–2020. Complementary search: English, abstract, free full text, full text, time limit 2020–2021		
	*CINAHL* Main search 2020–06-23 Supplementary search 2022-01-10		
# 1	Health personnel OR Health professional OR Students, health occupations	24,793	39,552
# 2	Ethics OR Ethics professional OR Morals OR Moral	30,348	6588
# 3	Simulation OR Patient simulation OR Teaching OR Teaching methods OR Teaching methods in nursing OR Learning Methods OR Education OR Learning	472,915	78,644
# 4	# 1 AND # 2 AND # 3	142	387
Search limiters	Main search: English, all text, abstract, full text, time limit 2010–2020. Complementary search: English, all text, abstract, full text, time limit 2020–2021		
	*PsycInfo* Main search 2020-06-23 Supplementary search 2022-01-10		
# 1	Health personnel OR Medical students OR Nursing students	210,242	11,098
# 2	Ethics OR Professional ethics OR Morale OR Morality OR Values OR Personal values	71,326,788	26,016
# 3	Simulation OR Simulation-Based Assessment OR Simulations Games OR Teaching OR Students Teaching OR Teaching Methods OR Team Teaching Methods OR Adaptive Learning OR Adult Learning OR Cooperative Learning OR Digital Game-Based Learning OR Experiential Learning OR Adult Education OR Medical Education OR Nursing Education	22,883,276	34,414
# 4	# 1 AND # 2 AND # 3	3443	422
Search limiters	Main search: English, peer reviewed, time limit 2010–2020. Complementary search: English, peer reviewed, time limit 2020–2021

Four of the authors (HA, AB, AS, and MH) independently screened all titles and abstracts, with the support of Rayyan QCRI, against the inclusion/exclusion criteria. The screening process consisted of two steps: (1) screening of articles identified in the main search and (2) screening of articles identified in the supplementary search. In the screening of articles identified in the main search, the blinded article selection in Rayyan QCRI indicated a 93% consensus between the authors with respect to the articles to exclude (n = 3811). After this, those articles for which there was no consensus regarding their inclusion (n = 287) were screened. Through discussions between the authors (HA, AB, AS, and MH), consensus was reached on which articles should then be excluded (n = 235). In the screening of articles identified in the supplementary search, the blinded article selection in Rayyan QCRI indicated a 95% consensus between the authors with respect to the articles to exclude (n = 953). After this, those articles for which there was no consensus regarding their inclusion (n = 42) were screened. Through discussions between the authors (HA, AB, AS, and MH), consensus was reached on which articles should then be excluded (n = 33). In total, 61 articles were selected for an additional full-text review. The articles were independently read in full by five of the authors (HA, AB, AS, MH, and AR) and then discussed, leading to an agreement to exclude 21 articles that did not meet the inclusion criteria. This led to 40 articles remaining for the quality assessment (see Fig. 1).

#### Stage 3: data evaluation

The quality assessment of the 40 articles was independently performed by two of the authors (HA and AB). A critical appraisal tool was used to score the articles on a four-graded scale (i.e., good, fair, poor, and very poor) [33]. The quality assessment consisted of two steps: (1) quality assessment of articles identified in the main search and (2) quality assessment of articles identified in the supplementary search. In the quality assessment of articles identified in the main search, there was consensus on the quality of 17 of the reviewed articles. However, there were different views on the quality assessment of 14 articles. Any discrepancies regarding authenticity, methodological quality, information value, and representativeness were considered, discussed, and resolved in the data evaluation process [34], leading to consensus between the authors regarding 11 articles pending between two adjacent grades: good–fair (n = 6), fair–poor (n = 3), and poor–very poor (n = 2). The authors’ quality assessment differed by more than one grade regarding three articles. However, even in these cases, the disagreement could be resolved through discussions between the two authors, after which a consensus was reached. In the quality assessment of articles identified in the supplementary search, there was consensus on the quality of 7 of the reviewed articles. However, there were different views on the quality assessment of 2 articles. Any discrepancies regarding authenticity, methodological quality, information value, and representativeness were considered, discussed, and resolved in the data evaluation process [34], leading to consensus between the authors regarding 2 articles pending between two adjacent grades good–fair. No articles were excluded due to a low-quality score. The characteristics of the included articles, as well as the quality scores, are presented in Table 2.

**Table 2:** Characteristics of the included articles

References and country	Design	Aim	Participants	Data collection	Analysis	Key findings	Quality scores
Baykara et al. [15], Turkey	Quantitative	To determining the effect of ethics training on fourth-year students of the nursing department on recognition of ethical violations experienced in the hospital and the development of ethical sensitivity	50 nursing students	Questionnaires Observation form	Analytical and descriptive statistics	Moral Sensitivity Questionnaire: The total pre-test scores in the experiment group were determined to be 93.88 + 13.57, and 91.48 + 17.59 in the control group. The total post-test scores in the experiment group were determined to be 89.24 + 15.90, and 97.72 + 19.91 for the control group. No significant differences were found between the pre- and post-tests. Observations: The experiment group performed more observations of ethical violations compared to students in the control group	Good
Blomberg et al. [45], Sweden	Qualitative	To explore and describe nursing students’ ethical reasoning and their supervisors’ experiences related to participation in clinical group supervision	17 nursing students	Interviews	Interpretative description	The form and content of clinical group supervision stimulated reflection and discussion of how to effectively handle situations with ethical aspects. Unethical situations were identified, and the process uncovered underlying caring actions	Good
Bowsher et al. [71], United Kingdom	Qualitative	To investigate the impact of a structured programme delivered online to support learning about ethical issues	19 medical students	Online transcripts	Thematic analysis	Five themes constitute the findings: (1) adopting a position on ethical issues without overt analysis, (2) presenting issues in terms of their effects on students’ ability to complete tasks, (3) describing local contexts and colleagues as ‘other’, (4) experiencing difficulties navigating between individual and structural issues, and (5) overestimating the impact of individual actions on structures and processes. While the students reflected on ethical issues, limited evidence of questioning or modification of their views was found	Good
Buxton et al. [54], USA	Qualitative	To describe an innovative use of interactive simulations to help midwifery students apply ethical principles in practice	20 midwifery students	Observations and online evaluation	Unknown	Ethical simulation can help improve the ability to reflect and deal with ethical issues. The simulation can be used as a formative experience	Poor
Chou et al. [52], Taiwan	Mixed methods	To explore how interprofessional education works in learning clinical ethics via problem-based learning and how different professions’ perspectives influence each other	45 medical students, 44 nursing students	Questionnaires	Analytical and descriptive statistics	Nursing students performed favourably on course engagement, caring, and communication, while medical students performed positively on issue identification and the life science aspect. Interprofessional group participation strengthened both professions’ performance better through the learning process	Fair
Gallagher et al. [55], United Kingdom	Qualitative	To describe experiences from an immersive 24-h simulation	18 nursing students	Focus groups interviews	Thematic analysis	Simulation is a way to promote ethics and to change participants’ perspective on caregiving and care-recipients	Fair
Gillam et al. [40], Australia	Qualitative	To introduce ethics educators to a narrative ethics approach in teaching	Part I: 12 educators Part II: 44 educators	Part I: Interviews Part II: Notes from small-group discussion, comments, and written feedback	Part I: Thematic analysis Part II: Unknown	Part I: Ethics educators should teach students about rational thinking. Part II: Ethics educators should teach students about how to manage emotions and handle unexpected and unwanted potential emotions	Poor
Grace et al. [39], USA	Case study	To describe a bioethics educational module	Medical residents, number of unknown participants	Unknown	Unknown	The five-box technique for bioethical decision-making included beneficence, nonmaleficence, autonomy, justice, and clinical integrity. Challenges have often been time based and related to the difficulty of predicting how long it will take to work through a case	Very poor
Harasym et al. [48], Taiwan	Theoretical paper	To discuss problem-based learning as an instructional strategy for teaching ethics	Not applicable	Not applicable	Not applicable	Problem-based learning is an effective instructional method for developing student ethical behaviours	Very poor
Hem et al. [43], Norway	Qualitative	To evaluate the significance of participating in systematic ethics reflection groups that focus on ethical challenges related to coercion	127 healthcare professionals	Focus group interviews	The analysis was inspired by the concept of ‘bricolage’	Most participants report positive experiences with participating in ethics reflection groups. The impact of the perceived lack of safety in reflection groups should not be underestimated. Sometimes, the method for ethical reflection was utilised in a rigid way	Good
Honkavuo [41], Finland	Qualitative	To deepen the understanding of ethics simulation in nursing education	6 nursing students	Interviews	Hermeneutic analysis	The nursing students’ narratives resulted in the meaning units: ethical being and ethos, nursing students’ formation process, bridge-building between theory and clinical practice, and teacher and ethics simulation	Good
Hsu [64], Taiwan	Quantitative	To assess nursing students’ satisfaction and attitudes as participants in a scenario-based learning (SBL) exercise and investigate the pedagogical application of SBL in a blended learning environment	99 nursing students	Questionnaires	Analytical and descriptive statistics	Students are relative positive with blended learning. Most of them (mean 4.10, SD 0.63) considered themselves good at using various learning strategies; a few (mean 2.55, SD 0.98) said they spent more time learning online than learning in class. Students reported that they ‘sometimes’ got involved in ethics discussions and determined appropriate action in ethically challenging situations. They ‘rarely’ helped patients make decisions on ethical issues	Fair
Kong et al. [62], United Kingdom	Qualitative	To evaluate a near-peer case-based undergraduate ethics teaching programme	32 medical students, 35 Foundation Doctors (FD)	Students: Anonymous feedback form FD: Face-to-face and email correspondence	Unknown	This programme provided students with an open and protected space in which they could reflect on their ethical behaviour. This programme was beneficial to FDs who were able to develop their own teaching and ethical reasoning skills and to reflect on the influence of the hidden curriculum on their own behaviour	Very poor
Kuhn et al. [69], Germany	Quantitative	To improve moral judgment, ethical reflection and strengthen individual resilience in value conflicts	13 medical students and young physicians	Web-based evaluation sheet	Descriptive statistics	Didactic concept with case conferences, discussion about ethics issues and lectures were considered helpful for dealing with ethical questions at the clinic. The format was also relevant for their later profession	Fair
Langlois et al. [61], Canada	Qualitative	To explore the experiences and learning of student health professionals engaged in an ethics module	91 health mentor programme students	Interviews	Thematic analysis	Five major themes emerged: (1) patient autonomy and expertise in care, (2) ethical complexity and its inevitable reality in the clinical practice setting, (3) patient advocacy as an essential component of day‑to‑day practice, (4) qualities of remarkable clinicians that informed personal ideals for future practice, and (5) patients’ perspectives on clinician error and how they enabled suggestions for improving future practice	Good
Lee et al. [63], Korea	Qualitative	To describe nursing students’ perspectives on and experiences of a case-centred approach to nursing ethics education using the four topics method	10 nursing students	Focus group discussion	Content analysis	Four themes emerged: 1) the importance of ethics education as perceived by nursing students,2) problems in current nursing ethics education, 3) the experience of case-centred nursing ethics education using the four topics approach, and 4) suggestions for improving nursing ethics education	Good
Lee et al. [73], Korea	Quantitative	To identify the effects of a nursing ethics seminar on the moral sensitivity and ethical behaviour of nurses working in a hospital setting	35 nurses	Questionnaires	Analytical and descriptive statistics	Moral sensitivity and unethical behaviour showed a negative correlation (r = 0.400, *p* < 0.05). After the ethics seminar, the experimental group’s moral sensitivity was not significantly increased (t = 1.039, *p* = 0.314). The experimental group’s mean scores of unethical behaviours at pre- and post-test were 12.59 and 9.47, respectively, indicating a statistically significant difference (t = 3.363, *p* = 0.004). There was no statistically significant difference in the mean score in both moral sensitivity and unethical behaviour at pre- and post-test in the control group	Good
Lillemoen et al. [44], Norway	Qualitative	To explore how ethics reflection in colleague groups was experienced and evaluated by the employees, facilitators, and service managers	Unknown	Focus group interviews Observations Written reports	Content analysis	Ethics reflection is a valuable measure to strengthen clinical practice. Ethics reflections have an impact on the climate of cooperation, not simply among the staff, but also with patients and their families. Ethics reflection is a process of change and both professional and personal development. Ethics reflection as a fragile established practice. This department has been characterised by high turnover and management changes	Good
Lin et al. [51], Taiwan	Quantitative	To pilot an interprofessional problem-based learning curriculum of clinical ethics and evaluate the curricular impact on interprofessional students’ attitudes and confidence in collaborative teamwork	36 medical students and nursing students	Questionnaires	Analytical and descriptive statistics	There was significant difference among different groups in terms of the students’ abilities and attitudes about ‘interprofessional communication and collaboration’ (*p* = 0.0184). The scores in the mixed group (37.58–3.26) were higher than those in the medical group (32.10–4.98). In terms of the students’ course satisfaction, the general satisfaction rating was around 79.41%. Most students (82.35%) considered the course effective in improving their understanding of clinical ethics	Good
Maddineshat et al. [65], Iran	Quantitative	To determine the impact of teaching ethics using games on moral sensitivity	30 nursing students	Questionnaires	Analytical statistics	The total score for moral sensitivity before and after the intervention showed significant changes (*p* = 0.02). Satisfaction with the ethics games for each session was higher than 4.4 out of 5. There was no significant trend between the means of the sessions (*p* = 0.66)	Fair
Martins et al. [67], Portugal	Quantitative	To determine whether bioethics education in nursing influences the level of moral competence and opinion of nursing students about three ethical dilemmas	122 nursing students	Questionnaires	Analytical and descriptive statistics	Nursing students showed a moral competence stagnation (1.2-point difference between the two assessments), although this did not reach statistical significance (*p* = 0.268). Regarding performance for each of the dilemmas, students showed an increase in performance for the worker's and judge's dilemmas and a sharp decrease in performance for the doctor's dilemma	Good
Martins et al. [68], Portugal	Quantitative	To investigate if the teaching of ethics can influence the moral competence of both medical and nursing students	263 nursing students and 70 medical students	Questionnaires	Analytical and descriptive statistics	For both nursing and medical students, C-score was lower after the attendance of the ethics curricular units, with a statistically significant decrease in the total score (from 21 to 19.5 on average; *p* = 0.046) for nursing students and a decrease not statistically significant for medical students (from 23.2 to 22 on average; *p* = 0.358). A multivariate analysis did not find any association between this decrease and gender, course, or age. The phenomenon of moral segmentation was observed, with better performance in the worker and judge dilemma, than in the doctor dilemma	Good
McCormick et al. [53], USA	Qualitative	Unknown	Medical social workers, number of unknown participants	Unknown	Unknown	Case studies could be used to illustrate ethical concepts and stimulate discussion, as well as to present new material to the participants	Poor
Mohammadi et al. [49], Iran	Quantitative	To examine the effect of bioethical principles teaching on moral attitude of paramedic emergency personnel in Kerman city of Iran	60 prehospital paramedic personnel	Questionnaires	Analytical and descriptive statistics	Ethical attitude means for both groups of control and intervention demonstrated that ethical attitude has meaningfully increased after the workshop	Good
Monteverde [50], Switzerland	Quantitative	To evaluate the feasibility and acceptance of the novel framework	58 nursing students	Questionnaires	Unknown	The framework was founded on problem-based learning. Students considered ethical theories—as taught within the proposed framework—as practically applicable, useful, and transferable to practice	Fair
Pandya et al. [58], India	Qualitative	To describe the experiences of using of narratives written by students in teaching	Unknown	Unknown	Unknown	The student writes a narrative from a patient’s perspective, based on real experiences. The sharing of narratives in the classroom, followed by discussion, can help to add different perspectives, and encourage both the narrator and the class to learn more about a scenario	Very poor
Parker et al. [38], Australia	Qualitative	To pilot an ethics teaching programme, with a focus on ethical issues that students are likely to encounter in their clinical education	47 medical students	Questionnaires	Analytical and descriptive statistics	Students feel a lack of experience and confidence in clinical ethics. Students liked the ‘real-life’ curriculum and the learning that comes from discussion of cases and personal experiences	Fair
Paton et al. [42], United Kingdom	Qualitative	To examine how storytelling and practical wisdom play integral roles in the medical ethics education of junior doctors	46 junior doctors	Interviews	Thematic analysis	Three major themes: (1) Learning medical ethics through storytelling, (2) Developing phronesis through storytelling and ‘story-listening’ and (3) Passing on/teaching phronesis through ‘phronesis narrative’	Fair
Sánchez-Izquierdo et al. [75], Spain	Quantitative	To develop a behavioural intervention to decrease paternalistic behaviours in formal caregivers and to increase care behaviours	118 health care professionals	Questionnaires	Analytical and descriptive statistics	Compared with the control group, caregivers in the behavioural intervention group displayed significantly lower paternalistic appraisals at the post-test and follow-up. Regarding the intervention group, caregivers at the post-test and follow-up showed a significantly greater occurrence of autonomist behaviours being promoted and lower paternalistic appraisal	Good
Savitha et al. [72], India	Mixed methods	To introduce an interactive and integrated ethics programme into the physiology course for first year medical students and to evaluate their perceptions	60 medical students	Questionnaires Focus group discussions Observer feedback	Descriptive statistics Content analysis	Students were exposed to a variety of ethical issues to reflect upon and stimulate their thoughts. All students felt that the programme was relevant to them	Fair
Schonfeld et al. [37], USA	Qualitative	To compare two teaching methods for ethics education	Medical students, number of unknown participants	Focus group interviews	Grounded theory	One theme: Student development and student engagement. Two categories: (1) understanding and (2) perspective	Fair
Shamim et al. [46], Saudi Arabia	Mixed methods	To develop and refine a contextually relevant approach to ethics education in the region of Saudi Arabia	46 medical students, 4 faculty members and 11 experts in the field	Focus group discussions Interviews Expert critique	Content analysis	Four main themes: 1) design features, 2) content, 3) teaching methods and 4) assessment. The results improved the design of the educational strategy	Good
Sherer et al. [36], China	Mixed methods	To examine ethics education programmes at three medical schools to understand their curricular content, teaching and learning methods, forms of assessment, and changes	407 medical students, 11 educators	Questionnaires Realistic cases for discussion	Analytical and descriptive statistics	Teaching methods: Lecture, small‑group discussion, and role play. Case-based learning: Appropriate when it involves a real‑world medical ethics issue. Inappropriate if it involves subject matter that students have not yet been exposed to	Poor
Silén et al. [60], Sweden	Quantitative	To investigate whether ethics rounds could improve the ethical climate	51 professionals from different professional groups	Questionnaires	Analytical and descriptive statistics	Ethics rounds did not result in significant changes in ethical climate	Good
Smith et al. [56], USA	Mixed methods	To evaluate high-fidelity patient simulation in teaching legal and ethical issues	221 nursing students	Questionnaires Scenario observation and debriefing	Analytical and descriptive statistics Content analysis	Simulation could support interaction, learning about real-life situations, and applying legal and ethical content in the scenario. Students need more preparation and information about the scenario and what they can do with the simulator. There were no significant differences between the students who played the nurse and those who played family members. However, students who played nurses were better at fulfilling their roles than students who played family members (*p* = .042)	Fair
Torabizadeh et al. [74], Iran	Qualitative	To evaluate the impacts of Socratic questioning on the moral reasoning of nursing students	103 nursing students	Questionnaires	Analytical and descriptive statistics	Both the teaching approaches improved the subjects’ moral reasoning; however, Socratic questioning proved to be more effective than lecturing	Good
Tsai et al. [70], Taiwan	Qualitative	To describe an ethical reasoning model and indicate how it can be used to foster moral and ethical behaviours	16 physicians	Interviews	Immersion and crystallisation approach	Ethical reasoning always began with information gathering, with or without verbalising the corresponding ethical problems. This was followed by decision making and, finally, by the production of an action plan. Ethical problems were described only when asked for; medical concerns were always raised first	Fair
Tsuruwaka et al. [57], Japan	Qualitative	To explore the effectiveness of learning the ethics of nursing practice using narrative writing	86 nursing students	Narrative scenes Comment sheets	Content analysis	Two core categories: (1) awareness of habits and trends in one’s own thoughts, and (2) awareness of organisational and administrative issues	Fair
Watts et al. [59], Australia	Quantitative	To evaluate Rural Ethics Ward Rounds	47 medical students	Questionnaires	Unknown	Students indicated that after participation, they were more aware of rural ethical issues (*p* < 0.01). Students found the openness of the sessions beneficial (91.7%) and were positive about the use of videoconferencing (86.6%)	Very poor
Yeom et al. [47], Korea	Quantitative	To examine the effects of nursing ethics education on moral sensitivity and critical thinking	70 medical students	Questionnaires	Analytical and descriptive statistics	There were no significant changes after the intervention in terms of moral sensitivity (*p* = 0.07) and critical thinking disposition (*p* = 0.44). There was a significant positive correlation between moral sensitivity and critical thinking before (*p* = .007) and after the intervention (*p* = .001)	Fair

#### Stage 4: data analysis

The data analysis was conducted by the first author. The findings were summarised and synthesised using a thematic analysis method [35] to identify the key themes that describe ethics education for healthcare professionals and students in clinical practice. This inductive approach also allowed us to answer the question regarding the design and content of ethics education and how ethics education could support the understanding and/or management of ethical problems in clinical practice, based on the available literature.

The analysis was conducted in the following six phases [35]: (1) reading and re-reading the included articles closely to become familiar with the data, (2) generating initial codes (228 codes in the present study) based on the information obtained from the included articles, (3) searching for themes, (4) reviewing themes, (5) defining and naming themes, and (6) producing a report where the findings are presented in terms of broad themes. The interpretation of the themes was discussed, and disagreement was resolved through discussion between the authors (HA, CF, AB, and AR) until a common understanding was reached.

## Results

Forty articles were included for review to explore the available literature regarding ethics education for healthcare professionals and students in clinical practice. The results showed a widespread international distribution of studies. Most of the studies were conducted in the United States (n = 5) and Taiwan (n = 5). When dividing the articles into continents, 17 were from Asia, 14 from Europe, six from North America, and three from Australia. Table 3 shows the key themes and sub-themes identified through the thematic analysis.

**Table 3:** Sub-themes and key themes identified in the review

Sub-themes	Key themes
Create conditions for learning	Making ethical competence learning possible
Design strategies for learning	
Interact with others	
Visualize attitudes and approaches	Having awareness of one’s own thoughts and perceptions
Experience emotional conditions	
Manage emotions and tensions	Doing right by the patient’s best interests
Manage different perspectives in a situation	

### Making ethical competence learning possible

Making ethical competence learning possible for managing ethical problems in clinical practice requires support. However, this support entails those certain conditions be met for learning in the organisation in which ethics education is conducted, including opportunities to plan the education. The design and content of education are governed by external structures and the way in which the learning objectives have been specified. To support learning, it is also important that education is designed to facilitate opportunities to receive and create meaning with respect to the information received, change one’s own values and attitudes, and determine the consequences of one’s own actions. Interaction with others is important since it can constitute a valuable source of knowledge, especially with respect to determining whether the individual healthcare professional or student has understood or done something correctly. Simultaneously, ethics education is influenced by both the healthcare professionals and the students who have different qualifications, expectations, and strategies for their learning.

The factors influencing the planning and organization of ethical education were discussed in 32 articles. Three sub-themes were identified: (1) creating conditions for learning, (2) designing strategies for learning, and (3) interacting with others.

#### Creating conditions for learning

A starting point for making ethical competence learning possible is to identify and shed light on the kinds of ethical problems that healthcare professionals and students in clinical practice are expected to be able to manage and to create conditions for this learning. Therefore, it is important that ethics education reflect the relevant conditions for ethical competence learning by using real work situations [36]. One way to create such conditions is to construct appropriate learning objectives that clearly describe what should be achieved in terms of knowledge, skills, approaches, and values to effectively manage ethical problems [37, 38]. However, the perception of what is relevant is influenced by healthcare professionals’ and students’ previous experiences of ethical problems in their everyday healthcare work. Limited experience entails a risk that the education will not be perceived as relevant, and that the educational content may be difficult to absorb [39].

Another condition that influences ethics education is the time available. Developing an ethical identity and creating meaning in discussions about ethical problems in everyday healthcare work takes time [37, 40]. Simultaneously, it might be difficult to predict how long, for example, group discussions may take to shed light on the various aspects of ethical problems [39]. There is thus a risk that the time will be too short and insufficient to finish the discussion, or that there may be too much time, thus causing the discussions to be perceived as less engaged [37]. Therefore, it is important that the time aspect be considered in the design of education.

Finally, it is essential to create conditions for psychological safety and confidence in ethics education, or, in other words, to enable opportunities to express opinions or make blunders without this leading to consequences for the participants [41]. Instead, trust between the participants should be emphasised and acknowledged in discussions about ethical problems in clinical practice [40, 42, 43]. Simultaneously, there is a risk that high staff turnover and frequent changes in management may limit opportunities for building trust through conversation [44]. Passive or absent healthcare professionals and students might also limit opportunities for establishing such trust, for example, in group discussions [45].

#### Design strategies for learning

Different design strategies make ethical competence learning possible, through which the healthcare professionals and students can be brought to ask questions, make comments, and talk about their previous knowledge or own experiences. Knowledge of, for example, ethical values can be gained through theoretical lectures and the reading of appropriate literature [46–48]. Simultaneously, it is valuable to design ethics education so that theoretical learning activities are integrated with practical ones and thereby provide an experience of real-life situations [46]. Skills can be practiced through workshops [49], case studies and problem-solving sessions [37, 43, 47, 48, 50–53]. Understanding one’s own values and attitudes can be facilitated through, for example, role-play or simulation activities [54–56], narratives [40, 57, 58], storytelling [42] and discussions in small groups [38, 43–45, 47, 59–62]. Small group discussions are appropriate when healthcare professionals or students are unwilling to stand out by asking questions or giving individual opinions in learning situations in which many people participate [63].

There are also different educational technologies to consider in the design of strategies for ethical competence learning. For example, the internet makes it easier to deliver lectures and carry out exercises [64], as well as to discuss issues in groups with digital aids [59]. This means that ethics education can take place in the form of internet-based education where video conferencing technique is used. This technique is valuable when using external educators in a rural setting for example in rural-based hospitals [59]. This technique is also useful to stimulate discussions with other healthcare professionals or students who are outside their regular workplaces [59]. However, a prerequisite for internet-based education is that the workplace has the required learning resources such as reliable internet connection and video equipment [64].

Ethics education needs to be built on strategies that optimise the ability to achieve the desired learning objectives [48]. To achieve these objectives, it may be necessary to choose different design strategies [36]. However, the strategy that best supports the development of a “professional self” is difficult to determine, for example, in terms of its ability to influence healthcare professionals’ and students’ capabilities for moral sensitivity [47, 65, 66] and critical thinking [47]. Nevertheless, support and learning activities do not necessarily promote ethical competence learning. Instead, these activities can also lead to stagnation in the development of ethical competence [67, 68].

#### Interacting with others

An open atmosphere and interaction between participants are important in ethics education when sensitive issues are discussed [69]. Sometimes, it is difficult to express one’s critical thoughts about ethical problems in everyday healthcare work, since relationships with others and cohesion between individuals can be affected and compromised [45, 57]. Simultaneously, there is a need for healthcare professionals and students to formulate their thoughts, feelings, and intentions about the ethical problems that they have observed themselves or heard about through colleagues [37, 38, 41, 43, 45]. Making ethical competence learning possible based on problem solving, interaction, and discussion of ethical problems in clinical practice can therefore be a support mechanism for healthcare professionals and students [37]. Learning together about issues that are perceived as ethically problematic can strengthen both the individual and their relationships with their colleagues [44, 52].

Simulation is a way of highlighting ethical problems that exist in interactions with other individuals, such as patients or family members [54]. Narrative groupwork is another way of highlighting and processing ethical problems [57]. Through a narrative, different perspectives can be made visible and lead to in-depth learning about ethically challenging work situations [58]. With group discussions, ethical problems can be viewed in different ways [59], which in turn can lead to improvements in dealing with such problems [44]. However, if group discussions are to lead to improvements, it is necessary that there be a willingness to discuss what is perceived as ethically problematic in everyday healthcare work [38, 45], as well as an interest in learning new approaches [37]. There is also a need for a welcoming climate in which the contradictions between different perceptions and attitudes can be balanced in a constructive way [43, 51].

### Having awareness of one’s own thoughts and perceptions

Ethical competence learning can help healthcare professionals and students in clinical practice direct their attention to ethical problems that they were not previously aware of. Such learning can involve unconscious attitudes, approaches, or emotions. These aspects influence how healthcare professionals and students react to ethical problems in everyday healthcare work.

The aspects that influence awareness of one’s own thoughts and perceptions were discussed in 22 articles in terms of both educational design and the content of ethics education. Two sub-themes were identified: (1) visualising attitudes and approaches, and (2) experiencing emotional conditions.

#### Visualising attitudes and approaches

Being aware of one’s own thoughts and perceptions in one’s attitudes and approaches to circumstances such as a certain illness, patient, or event influence what is perceived as an ethical problem in clinical practice [41, 70]. One way of designing ethics education that facilitates the visualisation of ethical problems is to use a narrative approach [40, 57, 58]. Using narrative writing, one’s own or others’ attitudes and approaches to everyday healthcare work situations where ethical problems occur can be made visible [57]. Examples of such ethical problems are when honesty and respect for the patient are not demonstrated, or when the establishment of trust in the care encounter is lacking [57].

Another way to visualise one’s own or others’ attitudes and approaches when designing ethics education is to use learning activities based on problems or scenarios [48, 51, 52, 54–56, 64]. This ethical competence learning focuses on challenging and realistic situations, such as conflicts regarding informed consent or cases where tensions arise between the patient’s wishes and needs in relation to professional norms [36]. Problem- or scenario-based learning stimulates healthcare professionals and students to learn and develop new understandings that allow them to manage ethical problems in their clinical practice [36]. Such learning could also create a means of engagement to discuss how ethical problems should be managed [64]. The visibility can also emerge by reserving time for ethical reflection and, in systematic forms, discussing ethical problems in everyday healthcare work [38, 43–45, 59, 70]. Attitudes towards a particular illness or patient, for example, govern our way of justifying the approaches used [70]. By highlighting how healthcare professionals and students think about and analyse their attitudes and approaches when designing ethics education, previous habits can be made visible and critically examined [44]. The visibility of attitudes and approaches promotes a process of change in one’s own thoughts and perceptions [43, 45]. However, it is essential to consider that attitudes and approaches are complex, developed over time, and strongly influenced by the perceptions of individuals who are close to the healthcare professionals and students undergoing training in healthcare professions [36, 48]. Accordingly, ethics education to support ethical competence learning does not always lead to a change in how ethical problems are managed in everyday clinical practice [71].

#### Experiencing emotional conditions

Awareness of one’s own or others’ emotions influences what is perceived as an ethical problem in everyday healthcare work. Healthcare professionals and students in clinical practice encounter a variety of ethical problems in which they are either actors or observers. Depending on the prevailing circumstances on site and at a given time, ethical problems, and their significance, as well as their relevance, can be experienced differently. When designing ethics education, real experiences, such as incidents that are ethically challenging and witnessed by healthcare professionals or students, can be used in ethical competence learning [58]. Group discussions make it possible for all participants to hear different interpretations and reflections on the same situation [38, 45]. Furthermore, such discussions can draw attention to situations where care and treatment have been experienced as unethical, such as when the patients’ concerns are not heard, or their needs are not met [61].

By imitating a realistic situation through simulation, healthcare professionals and students are given the opportunity to learn about real-life situations, apply ethical content in the situation, and experience different emotional states [56]. Educational content that highlights emotions, such as feelings of dependence, vulnerability, fear of abandonment, and a lack of control, gives healthcare professionals and students an opportunity to change their perspectives on factors such as caregiving and care-recipients [55]. Simulation can also be a way to raise awareness of other people’s ways of feeling and experiencing specific work situations, regardless of whether they play the role of professional, patient, or family member [56].

### Doing right by the patient’s best interests

Healthcare professionals and students in clinical practice are constantly faced with ethical problems related to patients, their significant others, colleagues, and the work organization. Dealing with such problems primarily involves reasoning about what is right and good to make decisions about what needs to be done in a specific situation. However, doing right based on the patient’s best interests can sometimes jeopardize the management of ethical problems since it could conflict with other patients’ interest, which may not be ethically acceptable or legally permitted.

Those aspects influencing healthcare professionals’ and students’ capabilities to do right by the patient’s best interests were discussed in 19 articles. Two sub-themes were identified: (1) managing emotions and tensions, and (2) managing different perspectives in the situation.

#### Managing emotions and tensions

Ethical problems can provoke strong emotions, such as anger, disapproval, and frustration [40]. These emotions, in turn, generate tensions, such as those between ethical values and legal principles in relation to how healthcare professionals and students in clinical practice perceive a particular situation [40, 51]. Therefore, it is essential that ethics education be designed to provide time and space for reflection. By reflecting together with others, thoughts and perceptions about these emotions and tensions can be verbalised [43, 72]. Ethics education should provide the opportunity to learn how to deal with emotions [40] and foster understanding of what is ethically ‘right’ or ‘wrong’ for the patient [45], which in turn influences the decisions made by healthcare professionals and students in clinical practice [51, 70]. Group discussion, for example in ethics seminar, is a way to reduce unethical behaviour [73]. There is, however, a difference between learning how to manage ethical problems in everyday healthcare work and how these problems are actually managed, since one’s own inabilities or limitations may influence the outcome [62].

#### Managing different perspectives on the situation

In everyday healthcare work, healthcare professionals and students face several challenges in determining how to ‘do the right thing’ in situations that arise in their contact with patients and their significant others. Ethical problems can arise when two perspectives, such as an ethical and a legal perspective, collide, as would be the case when there is conflict between what is perceived to be best for the patient and the patient’s right to self-determination [37]. There may also be a feeling of inadequacy in managing ethical problems in care situations [38] since there is rarely only one way to cope with the situation [51]. Therefore, ethics education needs to be designed in such a way that the content includes both medical and ethical reasoning when the care situation is to be resolved [70, 74].

The design of such training could consist of lectures that are combined with watching movies, playing games, and performing case analyses and group discussions [37, 47, 60, 65]. Through such training, an increased understanding of ethical problems can be gained [54, 72], for example, regarding the ways in which certain patients, events, and situations are to be viewed [37, 57, 65]. Ethical competence learning with a focus on ‘thinking ethics’ and problematising one’s own capabilities to judge and act can be an eye-opener for healthcare professionals and students [72, 75]. This can strengthen the capability to identify certain situations and provide examples of instances where ethical values and norms have been violated [66].

Even if the design and content of ethics education focus on thinking about critical ethics, this does not necessarily mean that the degree of critical ethics thinking is influenced [47]. Prerequisites for ethical competence learning of how to manage different perspectives and do right by the patient’s best interests are, among other things, that there is time for discussion, and that the educational content is perceived as useful [37]. It is also crucial that such learning be based on consideration and respect for different beliefs, so that ethical problems can be managed effectively in everyday healthcare work [43–45, 54].

## Discussion

Making ethical competence learning possible, having awareness of one’s own thoughts and perceptions, and doing right by the patient’s best interests are important aspects when seeking to increase the understanding and management of ethical problems in everyday healthcare work.

An important aspect emphasised in the present study is the need to create a psychosocial climate that allows healthcare professionals and students to feel safe. Previous knowledge reveals that feeling psychologically safe is important for engagement in educational activities, regardless of the context in which they are implemented [76]. Hence, it is important that educators use an approach that clarifies what psychological security in feedback conversations can look like [77]. To promote effective learning conditions in which healthcare professionals and students feel safe, educators need to encourage an open dialogue aimed at enhancing the implementation of the intended learning activity [76, 77].

The results present different designs and educational strategies for making ethical competence learning possible. In general, it is essential that educators develop course content that supports healthcare professionals and students in developing ethical competence in terms of their ethical decision-making ability and the moral courage to confront ethical dilemmas [78]. However, although ethical education might increase ethical sensitivity and the ability to detect an ethical problem, it is not obvious that education influences the development of ethical behaviour [79].

The results show how interaction with others is important since it constitutes a valuable source of knowledge; it also allows for the determination of whether the individual healthcare professional or student has understood or done something in an ethically defensible manner. Relationships between people constitute the foundation of ethics, and ethics is essential to the maintenance of relationships between two or more people [80].

Another critical aspect is the value of clinical experience. According to the results, limited experience poses a potential risk of not enabling healthcare professionals and students to absorb and contextually relate to the content of ethical education. However, previous research indicates that those with less clinical experience are more perceptive of ethical issues than more experienced colleagues, possibly counteracting the potential lack of experience [11].

The results underline the significance of attending to ethical problems that individual participants in ethics education may not already be aware of. This might be related to the fact that the patients, healthcare professionals, and students each have different and unique perspectives in caring encounters. To provide care based on the preferences of a specific patient, one needs insight into the patient’s lifeworld [81]. This might pose some challenges in designing and developing course content for ethics education.

Further, based on the present results, narrative approaches and realistic simulation are considered components that could influence ethical competence learning. Such learning should be based on the patient’s perspective to transform healthcare professionals’ and students’ tacit knowledge into explicit knowledge with support from reflective practice [82]. According to this, reflection with some theoretical depth grounded in caring science can contribute to a deeper understanding beyond that which is common in the clinical practice [83]. However, being aware of ethical problems—earlier not being aware of—raises new moral concerns among healthcare professionals and students. Thus, ethical education needs to be dynamically designed to capture different aspects of ethical problems.

The result highlights the importance of doing right by the patient’s best interests. Besides clinical competence, decisions regarding care and treatment also require ethical competence [3]. To do the right and good thing, an educational design that emphasises the healthcare professionals’ and students’ personal experiences, understanding, and views is required; such a skill can be cultivated, for example, through reflection [84]. Approaches such as moral case deliberation, ethics rounds, or discussion groups can be advantageously used to support ethical reflection [85]. At the same time, there are challenges regarding how ethical problems can be handled in clinical practice. Each problem and situation is unique, complex, and uncertain, since it can never be completely predicted. Therefore, doing right by the patient’s best interests may not necessarily only be about what to do in a specific situation; it can also be about scrutinising, interpreting, and processing other healthcare professionals’ and students’ knowledge, skills, and attitudes to ethical problems in clinical practice.

Doing right by the patient’s best interests also requires an educational design that provides space and time for reflection. Research indicates that the opportunity to share thoughts and obtain support from others, as well as from the organization, when ethical problems occur is considered helpful [86]. However, there are other factors that are essential for reflection. Space for reflection, for example, to create psychological safety is crucial for healthcare professionals and students to express themselves or make blunders without this leading to consequences. A hierarchical organizational climate influences sensitivity to ethical concerns, and a conformist work attitude could lead to an unwillingness to challenge routines in everyday clinical practice [86]. Time is also required to ensure that there is an opportunity for reflection. Without time, there is a risk that decision-making regarding ethical problems may become inconsistent [87].

### Methodological strengths and limitations

This study followed the recommendations for conducting and reporting the results of an integrative systematic review, and the researchers have strived to make the research process as transparent as possible, which is considered to have strengthened its reliability.

In this study, a broad literature search strategy was used to find as many articles as possible to answer the study aim and research questions. However, some issues may be encountered when conducting broad literature searches. One is that such a literature search likely leads to a greater number of irrelevant articles that match the search criteria. Another weakness is that it is time consuming to review a great number of articles. Accordingly, there is a risk that relevant articles may have been accidentally deleted, thereby weakening the validity of the study. However, this risk was partly managed by involving four of the authors in the screening process against the inclusion/exclusion criteria. The decision not to include “grey literature” can be considered a limitation as this may have affected the validity of the results.

Three available databases at a university in western Sweden were used. Since universities have different levels of licenses to access the contents of the databases, there is a risk that the search terms and search strings used in this study have failed to identify all articles on ethics education due to limited license agreements. Thus, there is a risk that some articles that are available in more extensive license agreements are not included in this literature review, which should be considered a limitation.

The decision not to include the perspective of those who supervise, and mediate ethics education could be seen as a weakness. However, it was a deliberate choice not to include the search term ‘educators’ based on the study aim and research questions. The requirements for educators can be different depending on whether the participants are students at a university or if they are healthcare professionals and are taught at their workplace. However, continued research on what competencies these educators should have in relation to supporting the learning and development of ethical competencies is important, and possibly points to a need for a systematic literature review that describes the educators’ competencies.

This study is limited to and focused on providing answers to questions regarding ethics education in various healthcare contexts in different countries. This is considered, on the one hand, to strengthen the validity and transferability of the results and, on the other hand, to limit the transferability of the results to contexts with similar cultural, economic, and social conditions, which are reflected in the included articles.

## Conclusion

This integrative systematic review provides insights into ethics education for healthcare professionals, students, and educators. The results show that ethical competence learning is essential when seeking to draw attention to and deal effectively with ethical problems. Healthcare professionals and students in clinical practice need a supportive learning environment in which they can experience a permissive climate for reflection on ethical challenges, conflicts, or dilemmas that influence everyday healthcare work. The design and course content of ethics education meant to increase the understanding and management of ethical problems in clinical practice may vary. However, regardless of the design or course content, educators need supportive conditions both on campus and in clinical practice to maximise opportunities to generate a high level of learning in ethics education.

Further studies on ethics education should be carried out. Comparative research, through which different educational designs can illuminate what provides the best possible learning process for managing ethical problems, would be valuable. Intervention studies aiming to maintain and protect the autonomy of patients with impaired decision-making capabilities may also be warranted. Another interesting area for further studies is about the educators’ and their competencies in ethics education with a special focus on the requirements if the participants are students at a university or if they are healthcare personnel and are taught at their workplace. Further studies could be used to develop healthcare professionals’ and students’ readiness and capabilities to recognise and respond appropriately when they encounter ethically problematic situations. This would, in turn, give healthcare professionals and students a sense of self-confidence and faith in their everyday clinical practice.

## Data Availability

The datasets used and/or analysed during the current study are available from the corresponding author upon reasonable request.
